# Supplementary material for: WBP2 inhibits microRNA biogenesis via interaction with the microprocessor complex
Source: Life Sci Alliance. 2021 Jun 11;4(7):e202101038. doi: 10.26508/lsa.202101038 (PMC8200299; doi:10.26508/lsa.202101038)
Supplement: Supplementary file 1 [file LSA-2021-01038_Supplemental_Data_1.docx]

**Antibodies**

Table 1 explains the details about the used antibodies.

**Plasmids and reporters**

V5-WBP2 and its PPxY mutants were generated as previously described (1). pcDNA3-V5-DGCR8 (DGCR8) was purchased from Addgene plasmid repository pCK-Flag-DGCR8 were kindly provided by Dr. V. Narry Kim (Seoul National University). To prepare the pri-miR-125b-1 and pri-miR-205 luciferase constructs, psiCHECK-2-TGFBR2-wt_3'UTR was purchased from Addgene. The insert sequence was cleaved out using XhoI and NotI restriction enzymes (New England Biolabs, United States). Primers harboring restriction enzyme sites were designed to amplify the, pri-miR-125b-1 (two-step nested PCR) and pri-miR-205 sequences, using isolated total RNA from MCF-7 cells. Following amplification, the insert sequences were digested with XhoI and NotI restriction enzymes (New England Biolabs, United States). Finally, pri-miR-125b-1 and pri-miR-205 were separately inserted downstream of Renilla gene using Rapid DNA ligation kit (Thermo Scientific, USA). The primers used for cloning are listed in **Table 2**.

**Small Interfering RNA (siRNA)**

Table 3 shows the siRNA sequences in detail.

**Probes and Primers**

The commercial mature miR-19a, miR-19b, miR-23a, miR-205 and U6 snRNA were purchased from Thermo Fisher Scientific Invitrogen (Carlsbad, California, USA). For protein-coding genes, and primary miRNA (pri-miRNA) and precursor miRNA (pre-miRNA) sequences, the quantitative PCR (qPCR) primers were purchased from IDT (Coralville, IA, USA) and are listed in Table 4.

1. Lim SK, Orhant-Prioux M, Toy W, Tan KY, Lim YP. Tyrosine phosphorylation of transcriptional coactivator WW-domain binding protein 2 regulates estrogen receptor α function in breast cancer via the Wnt pathway. The FASEB Journal. 2011;25(9):3004-18.
