# Supplementary material for: WBP2 inhibits microRNA biogenesis via interaction with the microprocessor complex
Source: Life Sci Alliance. 2021 Jun 11;4(7):e202101038. doi: 10.26508/lsa.202101038 (PMC8200299; doi:10.26508/lsa.202101038)
Supplement: Supplementary file 2 [file LSA-2021-01038_TableS1.docx]

**Table 1. List of antibodies used in the study.**

| **Antibody** | **Isotype** | **Source** | **MW (kDa)** |
| --- | --- | --- | --- |
| WBP2 (clone 4C8H10) | Mouse | EMD Millipore, USA | 37 |
| DGCR8 | Rabbit | Abcam, UK | 72 |
| Drosha | Rabbit | Abcam, UK | 140 |
| Rabbit | Abcam, UK | 68 | Rabbit |
| Rabbit | Proteintech, USA | 72, 80 | Rabbit |
| β-Tubulin | Mouse | Thermo Fisher Scientific, USA | 58 |
| V5 | Mouse | Thermo Fisher Scientific, USA | |
| Flag | Rabbit | Thermo Fisher Scientific, USA | |
| mouse (HRP) | Mouse | Thermo Fisher Scientific, USA | |
| rabbit (HRP) | Rabbit | Thermo Fisher Scientific, USA | |
