# Supplementary material for: WBP2 inhibits microRNA biogenesis via interaction with the microprocessor complex
Source: Life Sci Alliance. 2021 Jun 11;4(7):e202101038. doi: 10.26508/lsa.202101038 (PMC8200299; doi:10.26508/lsa.202101038)
Supplement: Supplementary file 3 [file LSA-2021-01038_TableS2.docx]

**Table 2. List of primers used for cloning.**

| **Plasmids** | **Primers (5′-3′)** |
| --- | --- |
| psiCHECK2-pri-miR-125b-1 | **F:** CCGCTCGAGCTTCAGTCATTTGTGCTAG  **R1:** GCGGCCGCTAACTGTGGAGTTTGAAAG  **R2:** TTTTCCTTTTGCGGCCGCTAACTG |
| psiCHECK2-pri-miR-205 | **F:** CCGCTCGAGGCAATTGCAGAACACC  **R:** TTTTCCTTTTGCGGCCGCGCTTTTCAGTAGACAAGC |
