# Supplementary material for: WBP2 inhibits microRNA biogenesis via interaction with the microprocessor complex
Source: Life Sci Alliance. 2021 Jun 11;4(7):e202101038. doi: 10.26508/lsa.202101038 (PMC8200299; doi:10.26508/lsa.202101038)
Supplement: Supplementary file 4 [file LSA-2021-01038_TableS3.docx]

**Table 3. List of siRNA sequences used in this study.**

| **Gene** | **Sequence** |
| --- | --- |
| **WBP2*** | 5′-CAGGAACUAGCAUUGUGGGACAUUA-3′ |
|  | 5′-CCUGGAUGCGCUAACAUUCACUCUU-3′ |
| **Drosha**** | 5′-GAAUGUCAUGUUAGCCUUUUACUGCAG-3 |
| **DGCR8*** | 5’-GGUGUAAGACAGAGAGUUGGAAA-3’ |
|  | 5’-GGAGACAUCGGACAAGAGUGUGAUU-3’ |
|  | 5’-UCAGCAAGCUCCAAGAGGAGAUGAA -3’ |
| **Universal Negative Ctrl**** | 5′-AUACGCGUAUUAUACGCGAUUAACGAC-3′ |

* siRNAs were obtained from Thermo Fisher Scientific Invitrogen (Carlsbad, California, USA).

** siRNAs were obtained from IDT (Coralville, IA, USA).
