# Supplementary material for: WBP2 inhibits microRNA biogenesis via interaction with the microprocessor complex
Source: Life Sci Alliance. 2021 Jun 11;4(7):e202101038. doi: 10.26508/lsa.202101038 (PMC8200299; doi:10.26508/lsa.202101038)
Supplement: Supplementary file 5 [file LSA-2021-01038_TableS4.docx]

**Table 4. List of the primers used in this study.**

| **Gene** | **Primer Sequence (5′-3′)** |
| --- | --- |
| **Pri-miR-23a** | **F:** TTCCAACCGACCCTGAGCTCT |
|  | **R:** ACTTAGCCACTGTGAACACGACTTGG |
| **Pre-miR-23a** | **F:** GGCCGGCTGGGGTTCCTG |
|  | **R:** TCGGTTGGAAATCCCTGGCAATG |
| **Pri-miR-19a/b** | **F:** AAGTGCTTATAGTGCAGGTAGTGTTT |
|  | **R:** ACCTGCAAAACTAACCATAGAACAGT |
| **Pre-miR-19a** | **F:** GCAGTCCTCTGTTAGTTTTGCA |
|  | **R:** GCAGGCCACCATCAGT |
| **Pre-miR-19b** | **F:** TTCTATGGTTAGTTTTGCAGGTTT |
|  | **R:** CACTACCACAGTCAGTTTTGCATGG |
| **Pri-miR-205** | **F:** GGAGGTTCCACATGGACTTATC |
|  | **R:** GCCTGTAGACACTGCATTACC |
| **Pre-miR-205** | **F:** TCCTTCATTCCACCGGAGT |
|  | **R:** GAACTTCACTCCACTGAAATCTGG |
| **Pre-U6 snRNA** | **F:** GTGCTCGCTTCGGCAGCA |
|  | **R:** GGAACGCTTCACGAATTTGCGTGTC |
| **GAPDH*** | **F:** TGCACCACCAACTGCTTAGC |
|  | **R:** GGCATGGACTGTGGTCATGAG |

* Glyceraldehyde 3-Phosphate Dehydrogenase
